# Event and Comment

**Published:** 1922-04

**Authors:** 


### DENTAL REGISTER

THE MAGAZINE OF DENTISTRY

Vol. LXXVI APRIL, 1922 No. 4

### EVENT AND COMMENT

**Value of Diagnosis as a Factor in Dental Health.**

We sometimes think there is a general breaking down of our dental health system, because we have so far failed to eradicate one or two of the diseases which most universally affects mankind, such as dental caries and periodontoclasia. Teeth have always been subject to decay and we have had periodontal diseases of all the soft tissues which embrace the teeth, and it seems in recent times the influence of these particular tissues and organs are not the local and peculiar affection we formerly believed them to be, but they are capable of producing most serious systemic diseases. In other words, the dentist is not only called upon to cure toothache, but also a large and increasing number of diseases that are closely allied with the general system. Many years ago some one banteringly asked Doctor W. H. Atkinson to tell him what was the cause of toothache. He gave what he thought was a sufficient reply when he said, "*show me the tooth,*" as though he could take a good look at the tooth and tell what was the trouble with it. Our present plans are quite as simple and sufficient for too many dentists, but we are gradually learning that to diagnose toothache is not the simple and sufficient answer to all present-day tooth ills. The radiograph has accomplished wonders to make clear the unseen causes of dental pathology and other impaired dental injuries. But with all these new evidences there are many more injuries and pathologic environments that we have only known when they have been revealed by serious systemic diseases which we do not even know they have even remotely influenced in any way bad health.

Do we realize what it means that dental caries is not a local disease any more, but that in its initial infection it is serious because it may and frequently does lead to the most serious systemic infections we know; such as neuralgia, arthritis and other vital organic infections?

Almost every infection, however insignificant at the first, eventually becomes not only consequential but may become a serious affection. We used to think that straightening children's malposed teeth was a question only of physics and mechanics, but the orthodontist is fast becoming a scientist with an expanding interest in the knowledge and skill of the medical expert internest. The same may be cited of oral prophylaxis or the dental hygienist, who a few years ago thought his job was to clean away from the teeth calcular deposits and polish away discolorations that were only offensive to sight, but these were not seriously considered questions of ill-health, and liable to infect the entire body in any serious manner. We are now finding out that our crown and bridge work must be changed because it is a source of systemic ill-health. Even plate prosthesis is being studied to determine what can and should be done to give better health for such mechanical appliances, because unless properly constructed, however artistically made, they may be serious factors in the health propaganda. In fact the dental profession is undergoing a serious examination to find out just what can be done that will make for better health, in the way of correcting by artistic endeavors proper technical appliances. It is in reality a more serious problem that confronts what has been for a long time only a technical incident in our American system of dental practice. It is now imperative that the dentist shall make efficient inspections of all teeth, that he may assume his full obligation as an important guardian of the public's health.

